# Supplementary material for: Shared genetic etiology between obsessive-compulsive disorder, obsessive-compulsive symptoms in the population, and insulin signaling
Source: Transl Psychiatry. 2020 Apr 27;10:121. doi: 10.1038/s41398-020-0793-y (PMC7186226; doi:10.1038/s41398-020-0793-y)
Supplement: Supplementary file 3 — Supplementary Table 2 [file 41398_2020_793_MOESM3_ESM.docx]

| **Supplementary Table 2.** Content of two novel OCS factors that could be compiled from the Toronto Obsessive-Compulsive Scale (TOCS) questions and that are similar in the questions they contain to the two factors (‘symmetry/counting/ordering’ and ‘contamination/cleaning’) from the Philadelphia Neurodevelopmental Cohort (PNC) data. |
| --- |
| **Factor Symmetry/counting/ordering _TOCS_** |
| TOCS items: |
| “Do Certain”: Needs to do certain things (e.g., counting steps) |
| “Checks”: Checks things |
| “Count”: Needs to count objects |
| “Symmetry”: Needs things to be symmetrical |
| \| “Interfere”: Gets upset when people rearrange things \| \| --- \| |
| “Repeat”: Repeats actions until just right |
| “Not Exactly”: Worries if something done not exactly right |
|  |
| **Factor Contamination/cleaning _TOCS_** |
| TOCS items: |
| “Dirt”: Hates dirt/dirty things |
| “Germs”: Worries about germs |
| “Wash”: Needs to wash hands |
| “Clean”: Worries about being clean |
|  |
