# Supplementary material for: Shared genetic etiology between obsessive-compulsive disorder, obsessive-compulsive symptoms in the population, and insulin signaling
Source: Transl Psychiatry. 2020 Apr 27;10:121. doi: 10.1038/s41398-020-0793-y (PMC7186226; doi:10.1038/s41398-020-0793-y)
Supplement: Supplementary file 4 — Supplementary Table 3 [file 41398_2020_793_MOESM4_ESM.docx]

| **Supplementary Table 3.** MAGMA-based gene-set analysis for the 51 CNS insulin signaling genes extracted from our earlier-defined OCD landscape. P-values were considered signiﬁcant if they exceeded a Bonferroni-corrected threshold accounting for the number of phenotypes tested (P < 0.05/7 tests (total OCS score and six OCS factors) = 0.00714). | |
| --- | --- |
| Trait | P-value |
| Total OCS score | 0.76762 |
| Impairment | 0.98503 |
| **Symmetry/counting/ordering** | **0.00408** |
| Contamination/cleaning | 0.33586 |
| Aggressive taboo thoughts | 0.30643 |
| Guilty taboo thoughts | 0.16893 |
| Distress | 0.32738 |
