# Supplementary material for: Shared genetic etiology between obsessive-compulsive disorder, obsessive-compulsive symptoms in the population, and insulin signaling
Source: Transl Psychiatry. 2020 Apr 27;10:121. doi: 10.1038/s41398-020-0793-y (PMC7186226; doi:10.1038/s41398-020-0793-y)
Supplement: Supplementary file 5 — Supplementary Table 4 [file 41398_2020_793_MOESM5_ESM.docx]

| **Supplementary Table 4.** Gene-wide association results for 51 unique genes from the OCD landscape and the OCS factors ‘symmetry/counting/ordering’ # | | | |
| --- | --- | --- | --- |
| Gene | P-value | Gene | P-value |
| *ADD3* | 0.2045 | *LNX1* | 0.4954 |
| *ARHGAP15* | 0.5126 | *MEIS2* | 0.4562 |
| *BDNF* | 0.5325 | *MTUS2* | 0.2716 |
| *BTBD3* | 0.0080 | *NOS1* | 0.2508 |
| *CCNC* | 0.1823 | *NSG2* | 0.9420 |
| *CYTIP* | 0.1470 | *PDE4D* | 0.7043 |
| *DCC* | 0.3287 | *PRDM13* | 0.4412 |
| *DLGAP1* | 0.6954 | *RACGAP1* | 0.7330 |
| *DLGAP3* | 0.0465 | *REXO1* | 0.2719 |
| *DNAI1* | 0.5131 | *SEMA4D* | 0.1606 |
| *DOCK1* | 0.5564 | *SERPINH1* | 0.2660 |
| *EBF2* | 0.4170 | *SLC1A1* | 0.1345 |
| *EFNA5* | 0.1995 | *SLIT3* | 0.5258 |
| *EREG* | 0.1279 | *SLITRK5* | 0.9651 |
| *FKBP1A* | 0.6049 | *SORBS1* | 0.1688 |
| *GJD2* | 0.1083 | *TBP* | 0.2024 |
| *GNRH1* | 0.1992 | *TFDP2* | 0.1525 |
| *GRIN2B* | 0.0217 | *TMEM252* | 0.1308 |
| *HOXB8* | 0.3795 | *TNF* | 0.7841 |
| *HTR1B* | 0.8224 | *TRIOBP* | 0.8752 |
| *IGF1* | 0.0085 | *TSPAN14* | 0.2407 |
| *IGF1R* | 0.7069 | *TXNL1* | 0.8934 |
| *IRS2* | 0.5154 | *UBL3* | 0.0890 |
| *ITGA9* | 0.8512 | *ZBTB43* | 0.5905 |
| *KCNB2* | 0.3086 | *ZFP64* | 0.5493 |
| *KCNQ1* | 0.8696 |  |  |

^#^Note: Shown in this table are the gene-wide results from the MAGMA analyses of the 51 genes from the molecular landscape of OCD and the OCS factor ‘symmetry/counting/ordering’. None of the individual genes reached the Bonferroni-corrected P-value threshold of significance (P=0.05/51=0.00098).
