# Supplementary material for: Shared genetic etiology between obsessive-compulsive disorder, obsessive-compulsive symptoms in the population, and insulin signaling
Source: Transl Psychiatry. 2020 Apr 27;10:121. doi: 10.1038/s41398-020-0793-y (PMC7186226; doi:10.1038/s41398-020-0793-y)
Supplement: Supplementary file 7 — Supplementary Figure 1A [file 41398_2020_793_MOESM7_ESM.pdf]

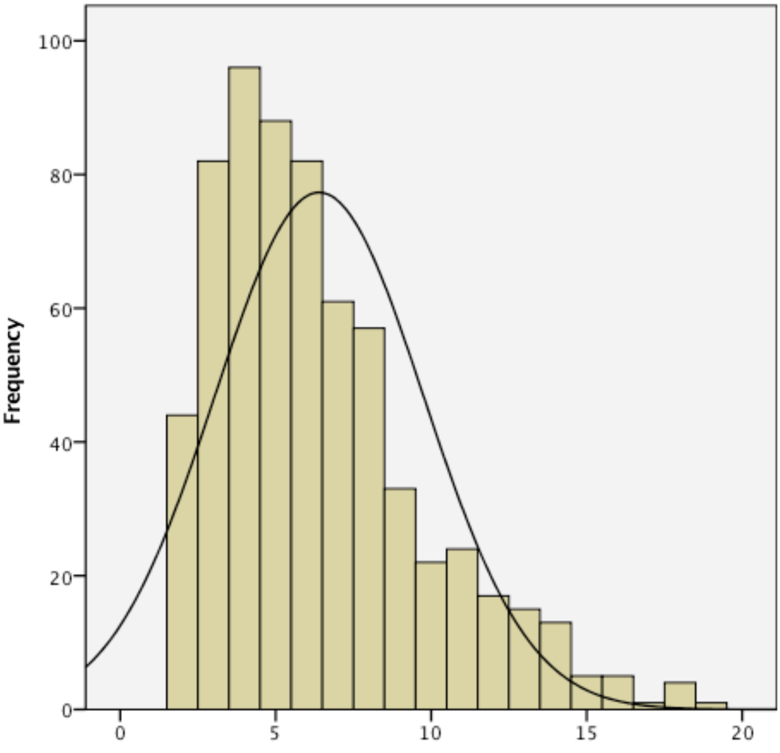

Supplementary Figure 1A. Histogram showing the distribution of the total OCS score in 650 children and adolescents aged 8-21 in the Philadelphia Neurodevelopmental Cohort (288 males and 362 females).
