# Supplementary material for: Shared genetic etiology between obsessive-compulsive disorder, obsessive-compulsive symptoms in the population, and insulin signaling
Source: Transl Psychiatry. 2020 Apr 27;10:121. doi: 10.1038/s41398-020-0793-y (PMC7186226; doi:10.1038/s41398-020-0793-y)
Supplement: Supplementary file 8 — Supplementary Figure 1B [file 41398_2020_793_MOESM8_ESM.pdf]

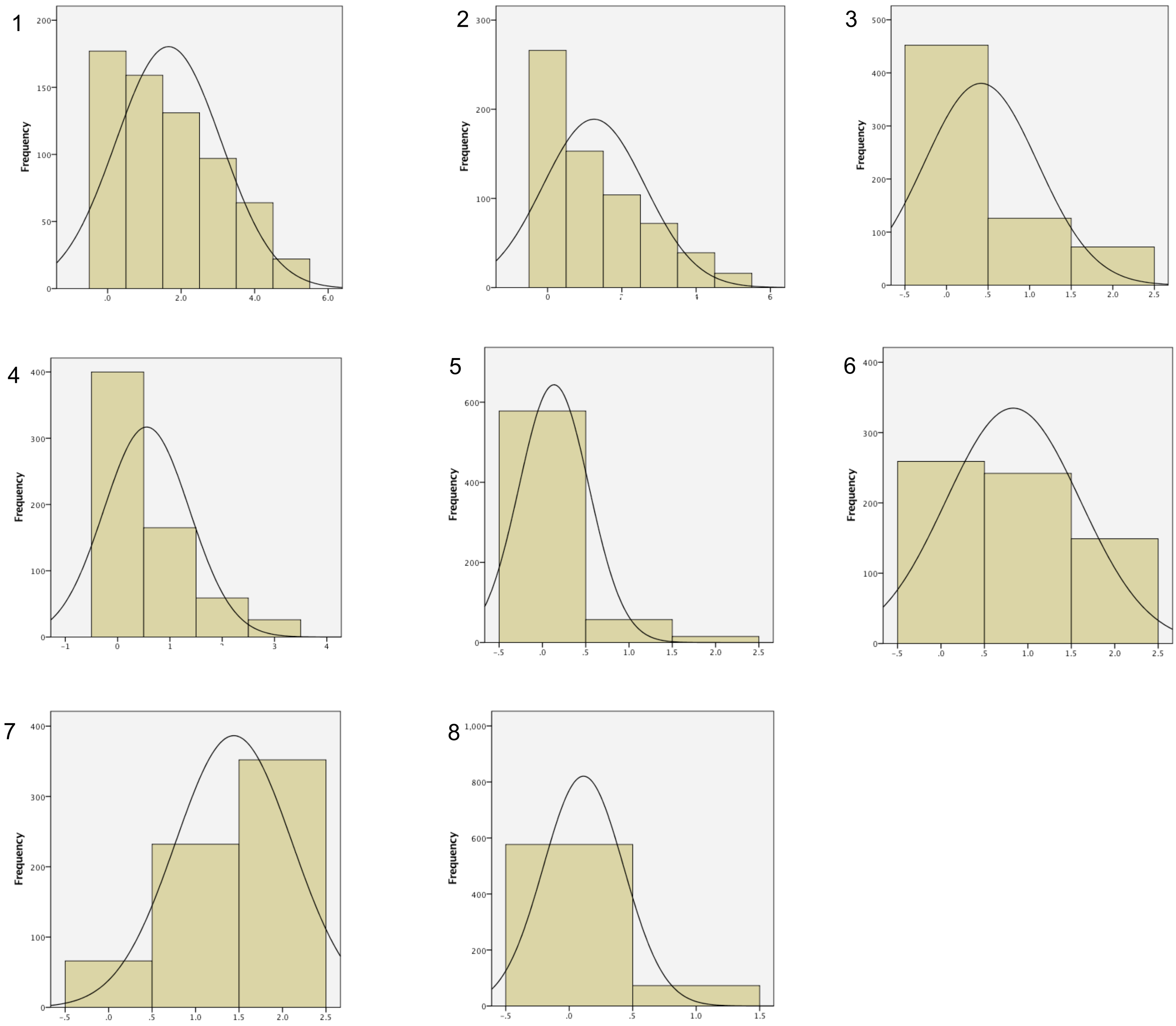

Supplementary Figure 1B. Histograms showing the distributions of the scores on eight OCS factors - that add up to the total OCS score - in 650 children and adolescents aged 8-21 in the Philadelphia Neurodevelopmental Cohort (288 males and 362 females): 1 'Impairment', 2 'Symmetry/counting/ordering', 3 'Contamination/cleaning', 4 'Aggressive taboo thoughts', 5 'Repetition', 6 'Guilty taboo thoughts, 7 'Distress', and 8 'Religious taboo thoughts'.
