# Supplementary material for: Shared genetic etiology between obsessive-compulsive disorder, obsessive-compulsive symptoms in the population, and insulin signaling
Source: Transl Psychiatry. 2020 Apr 27;10:121. doi: 10.1038/s41398-020-0793-y (PMC7186226; doi:10.1038/s41398-020-0793-y)
Supplement: Supplementary file 9 — Supplementary Figure 2 [file 41398_2020_793_MOESM9_ESM.pdf]

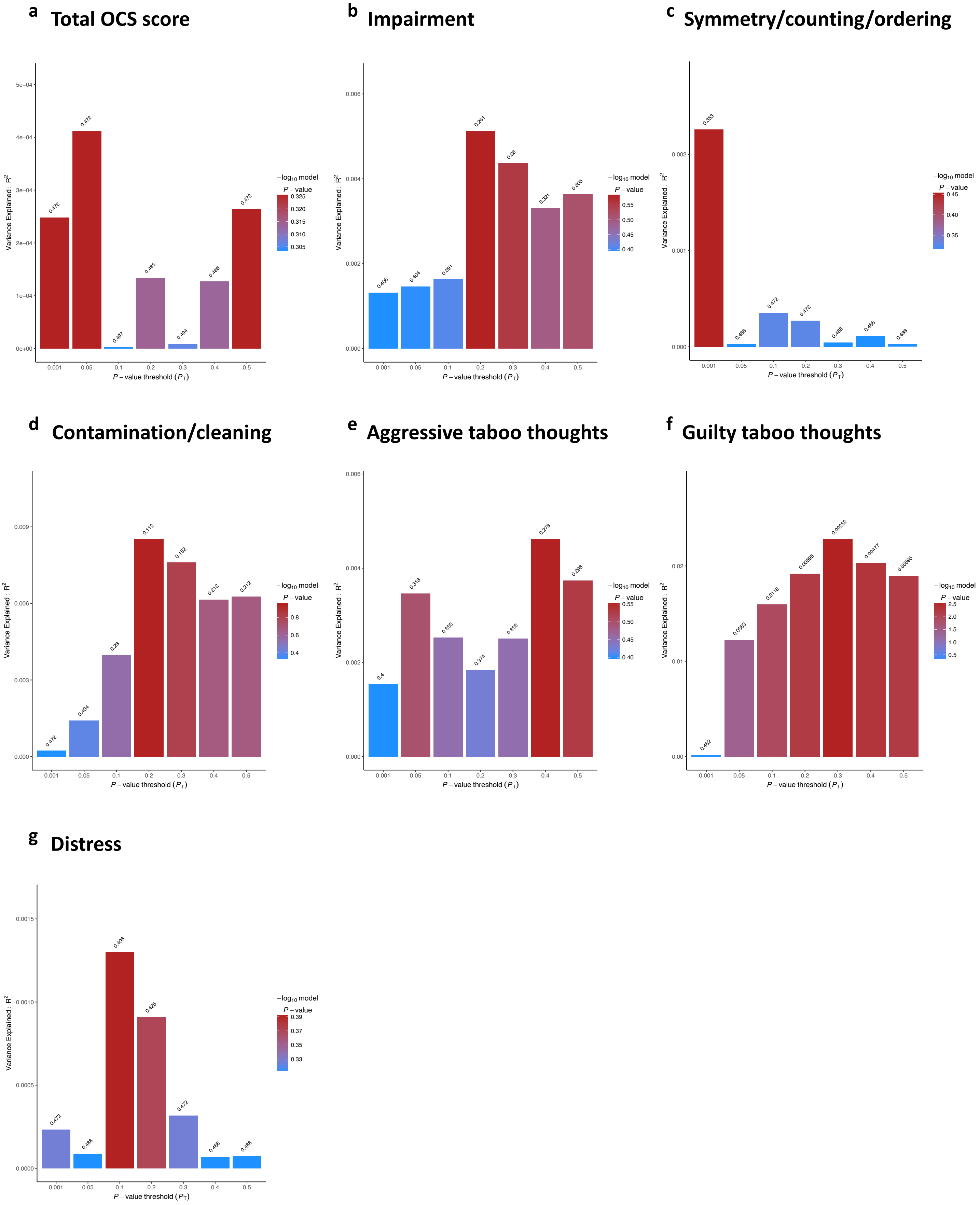

Supplementary Figure 2. Bar plots from PRSice showing results at seven broad P-value thresholds (P_T) for shared genetic etiology between obsessive-compulsive disorder (OCD) and the total obsessive-compulsive symptom (OCS) score as well as six OCS factors (a–g) (see Material and Methods). The numbers above the bars indicate the P-values for shared genetic etiology, and these P-values were corrected using the Benjamini-Hochberg false discovery rate method.
