# Supplementary material for: Shared genetic etiology between obsessive-compulsive disorder, obsessive-compulsive symptoms in the population, and insulin signaling
Source: Transl Psychiatry. 2020 Apr 27;10:121. doi: 10.1038/s41398-020-0793-y (PMC7186226; doi:10.1038/s41398-020-0793-y)
Supplement: Supplementary file 15 — Supplementary Figure 4A [file 41398_2020_793_MOESM15_ESM.pdf]

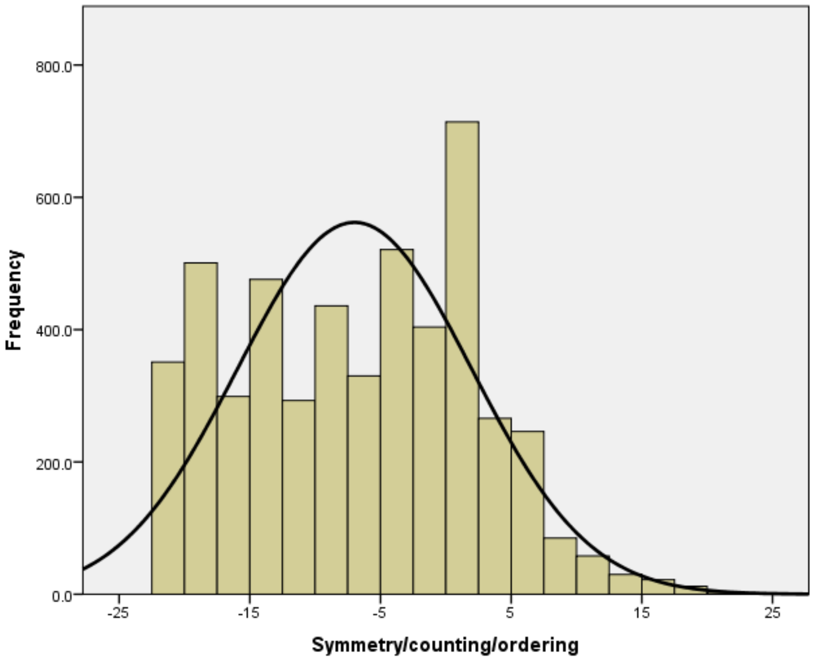

Supplementary Figure 4A. Histogram showing the distribution of the 'symmetry/counting/ordering' score in 5047 children and adolescents aged 6-17 in the Spit for Science Cohort.
