# Supplementary material for: Shared genetic etiology between obsessive-compulsive disorder, obsessive-compulsive symptoms in the population, and insulin signaling
Source: Transl Psychiatry. 2020 Apr 27;10:121. doi: 10.1038/s41398-020-0793-y (PMC7186226; doi:10.1038/s41398-020-0793-y)
Supplement: Supplementary file 18 — Supplementary Figure 5B [file 41398_2020_793_MOESM18_ESM.pdf]

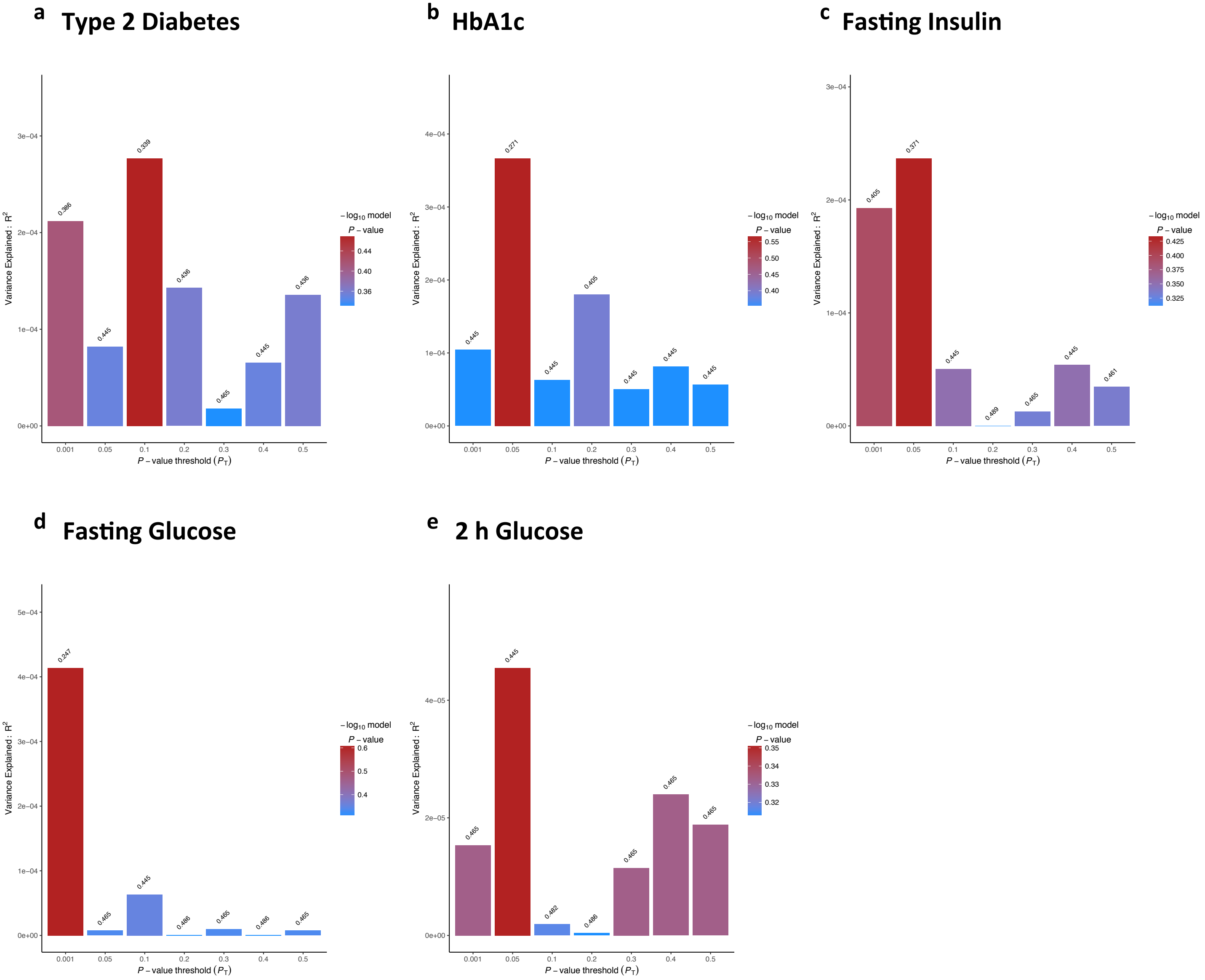

Supplementary Figure 5B. Bar plots from PRSice showing results at seven broad P-value thresholds (P_T) for shared genetic etiology between five peripheral insulin signaling-related traits (Type 2 Diabetes, HbA1C blood levels and blood levels of fasting insulin, fasting glucose and 2h Glucose) and TOCS factor ‘symmetry/counting/ordering’ (a–e) (see Methods). The numbers above the bars indicate the P-values for shared genetic etiology, and these P-values were corrected using the Benjamini-Hochberg false discovery rate method.
